# A Brief Notice of Two Cases Occurring in the Surgical Wards of the Pennsylvania Hospital

**Published:** 1841-04-10

**Authors:** G. W. Norris

**Affiliations:** Surgeon


### MEDICAL EXAMINER.

DEVOTED TO MEDICINE, SURGERY, AND THE COLLATERAL SCIENCES.

No. 15.] PHILADELPHIA, SATURDAY, APRIL 10, 1841. [Vol. IV.

### ORIGINAL COMMUNICATIONS.

*A Brief Notice of two Cases occurring in the Surgical Wards of the Pennsylvania Hospital.* By G. W. Norris, M. D., Surgeon. Reported by *Alfred Stillé*, M. D., late Resident Physician.

S. F. C., æt. twenty-one, was admitted into the Pennsylvania Hospital, Oct. 21st, 1839, having phagedenic ulcers of the prepuce and phimosis, of three days' standing. He was discharged, cured, on the 16th of the following month. Sometime during the subsequent winter he contracted a gonorrhœa, which, from neglect or improper treatment, remained uncured in the summer of 1840. At the commencement of the fall of that year he applied to one of those reckless triflers with human life, known as Thompsonian physicians, who gave him a mixture to be injected into the urethra. After using it the patient complained of intense heat and pain about the genitals, insufferable scalding of his urine, and there soon after appeared a tumour in the perineum, which increased in size until it reached that of a hen's egg, and caused the greatest difficulty, as well as severe pain in urinating. In this condition C. entered the hospital, Oct. 16th, 1840; when the tumour in the perineum was found to be an abscess, which, on being opened, discharged four or five ounces of pus, and left a fistulous opening passing into the urethra. The inflammation of this latter was still active; a purulent discharge escaped from it; the passage of urine through it created intense suffering, the less easily borne, because the bladder was unable to retain its contents more than a half an hour at a time. By means of emollient drinks, cataplasms, rest, and diet, these symptoms were in a good degree moderated; but the micturition persisted. Suddenly one day early in December, the patient was seized with a severe pain passing from the perineum, towards the left lumbar region, accompanied with a chill, and fever. Pressure over the left kidney, and along the course of its ureter, was painful, the abdomen became distended with gas, the quantity of urine less, and of a deeper colour, and very soon it deposited on standing, a large quantity of pus. In the left lumbar region could be felt a greater resistance than elsewhere in the abdomen, there was a greater roundness and prominence of this part, and percussion over it was generally flat. The symptoms of acute nephritis having been overcome, the patient continued during a month to improve in strength and flesh, but at the end of that period was attacked one night with a fit of coughing and suffocation, during which he discharged from his mouth nearly a pint of pus. The signs of a tumour in the left lumbar region at once became less marked, the discharge of pus with the urine diminished, and at last ceased, and there was added a frequent paroxysmal cough, with muco-purulent expectoration. On ausculting the lungs, the natural sounds of respiration were found unaltered in the right one, but in the left, the lower third behind, dull on percussion, offered to the ear a loose ronchus almost like gurgling, there were bronchial breathing and voice at the root, and a slight mucous ronchus there, and at the summit of this lung. By degrees the flatness on percussion disappeared, the expectoration became rather less abundant, and the general condition of the patient remained nearly stationary until the beginning of the month of February, when his cough became more frequent and violent, his body grew thinner, his legs œdematous, and his strength failed him entirely. Towards the middle of the month a tumour appeared in the left groin directly over the psoas tendon; it grew red, pointed, and burst; discharging nearly a pint of pus and serum. From that moment the expectoration ceased, nor was it renewed until within a few days of the patient's death, and then but very scantily.

C. died on the 10th of March. His body was examined, and the following lesions discovered. The bladder was very small, its coat thick and hard, its mucous membrane rugose and firm. The left ureter was larger than the right, its canal being smaller, its walls thicker, and very firm, its internal membrane injected with red vessels and bathed with pus. The corresponding kidney was small and tightly bound in its position by false membranes; it was filled with abscesses, some of which communicated with a large purulent collection situated between the kidney and the psoas muscle, passing downwards along the latter to the opening as mentioned as having taken place in the groin, and upwards by the side of the crura of the diaphragm, penetrating this muscle and forming an enlargement between it and the base of the lung, which was elsewhere adherent to the diaphragm. In the portion of the lung, bathed by the contents of the abscess, was a rounded but rough opening, about a quarter of an inch in diameter, in connection with extensive abscesses running in every direction throughout the lung, and in their turn opening into the bronchia.

In the lower lobe there were a great many isolated tubercles some of them as large as grains of rice, and in the upper lobe several cavities, having many of the characters of those formed by the softening and evacuation of tubercles; others again were not lined by a distinct false membrane, and were filled with a turbid liquid resembling a mixture of pus and serum. One or two of these cavities existed in the right lung; a few crude tubercles were scattered throughout it. The right kidney was about twice as large as the left; its structure appeared to be healthy.

The posterior spinous processes of the left ilium were found to be carious; they, and the bone for an inch around them, were divested of periosteum, and in contact with the pus contained in the abscess already described.

It rarely happens that the progress of an internal disease can be so distinctly traced, during life, as in the present instance, and it perhaps is equally uncommon to see an inflammation travelling speedily onward from organ to organ, until at last death is the consequence, not so much of any one alteration of structure, as of a series of formidable lesions. This chain had, in the present case, its first link in the injudicious prescription of an ignorant impostor: he probably neither knew nor cared about the bladder which lay beyond the urethra he was treating so heroically, nor about the ureter, the kidney, the lung, which were destined successively to be destroyed by his pernicious drugs. His ignorance may palliate, but cannot excuse his crime; *we*, who profess to know more of the relations, the functions, and the susceptibilities of those organs, may learn a lesson from his error—that of caution in the use of stimulating injections in inflammations of the urethra. When prescribed with judgment, and used with extreme prudence, they are doubtless valuable agents, but ought not to be trusted to an inexperienced or a careless hand.

A. M., æt. forty-three, a seaman, of good constitution, was affected with quotidian intermittent fever, of a malignant type, in September, 1840; he had only three paroxysms, when the disease appeared to be arrested, and the patient returned to his business. But every fourteenth day thereafter he experienced a chill, with fever and sweating, more or less marked. One of these periods fell upon March 2d, 1841. M. had had a scrotal hernia of the left side, from childhood, and generally wore a truss. On March 1st, while at stool, his truss being in its place, M. felt his hernia escape, and when he tried to replace it, found himself unable to succeed. The assistance of two physicians was immediately procured; and the taxis, together with the use of purgative medicines, and depletion, was employed by them, from about 4 o'clock, P. M., on the 1st, until 10 A. M., on the 2d March, when the patient was brought to the hospital. The hernial tumour extended from the upper part of the left side of the scrotum to near the position of the internal abdominal ring; it was regularly rounded in form, and may have measured about three inches long by two and a half inches wide; it was sore upon pressure, which circumstance the patient attributed to its having been so frequently handled; on percussion the upper third was found to be flat, the lower two-thirds resonant. There was no tenderness of the abdomen; but little vomiting of mucus or bile; no chilliness; the pulse was slow, soft, and moderately full; and the patient complained of nothing but a slight soreness in the left groin. Efforts were renewed to effect a reduction of the hernia; warm applications to the part; the warm bath till faintness came on,—then pounded ice over the tumour, with purgative medicines by the mouth and rectum, were all tried in turn, but unavailingly. The general symptoms, however, were not urgent, and nothing except an occasional hiccough showed that the patient was suffering. On the 3d March, no material change had taken place; M. had passed a comfortable night; the tumour retained its previous characters; there was a little more inclination to vomit, and the prostration was more marked. The tobacco enema was now resorted to; soon after its administration, the pulse became more frequent and feeble, and the patient complained of a strange giddiness in his head, and a feeling of weakness. The taxis was again employed with no better result than before. In consultation it was decided to operate; and the dangers being frankly stated to the patient, he gave his consent.

Dr. Norris accordingly performed the operation in the presence of the medical students, about noon, and with some difficulty succeeded in reducing the hernia, but not until he had first restored to the abdomen a portion of mesocolon, which, descending with the intestine, formed an impassable pad immediately without the abdominal ring. The hernia consisted of this portion of mesocolon, and of a loop of the corresponding intestine, which was slightly injected, but neither gangrenous nor of a dark colour; it was distended by air. There was a trifling hæmorrhage from the *arteria ad cutem abdominis*, which was at once arrested by the application of a ligature. In other respects there was nothing peculiar about the operation, except the length of time it required for its performance, owing to the resistance offered by the mesocolon to the return of the other portion of the hernia.

The patient was put to bed, and continued during the afternoon free from pain; his bowels were more freely opened than before; but his pulse became more frequent, his expression more anxious, and there was a constant hiccough at intervals of four or five minutes. During the night there was an increase of pain in the left groin, and some soreness on pressure on the corresponding side of the abdomen, with considerable restlessness and unwillingness of the patient to lie on his back. On the morning of the 4th, the pulse was more frequent, (about 140,) very feeble, the general prostration, hiccough, contraction of the features, and jactitation more marked, the abdominal tenderness moderate, with slight meteorism; there were great thirst, a little vomiting, and copious dejections, procured by enemata. No important symptom was added to those already enumerated; but the patient continued to sink throughout the day, and died about 10 o'clock, P. M.

An examination of the body was made about twelve hours after death, when the peritoneum in the neighbourhood of the strangulated portion of the intestine was found moderately injected: but there was no fluid in the cavity of the abdomen, nor any recent false membrane, nor any gangrene. There did not appear to be any disorganization whatever in that part of the colon which had formed the hernia, only the diameter of the intestine was sensibly less at this point than either above or below it.

What was the cause of death in this case? The symptoms were not threatening before the operation, which the character of the hernia, however, rendered imperatively necessary; and the lesions found after death are not sufficient, we think, to explain the fatal termination. Might the state of the system, at a time when it was threatened with a paroxysm of intermittent fever, have been such as to render death the consequence of causes, which, under other circumstances, would have been incapable of producing such a result?

